# Analysis of Prenatal Diagnosis and Pregnancy Decisions of 767 Singleton Pregnancies With Positive Prenatal Cell-Free DNA Screening Results in Southwest China

**DOI:** 10.1155/jp/8877014

**Published:** 2025-11-12

**Authors:** Yun Chen, Yunli Lai, Fuben Xu, Yanqing Tang, Fanglu Wei, Lintao Meng, Haisong Qin, Jiasun Su, Weijia Sun, Yiping Shen, Hongwei Wei

**Affiliations:** ^1^Genetic and Metabolic Central Laboratory, Maternal and Child Health Hospital of Guangxi Zhuang Autonomous Region, Nanning, Guangxi, China; ^2^Birth Defects Prevention and Control Institute of Guangxi Zhuang Autonomous Region, Nanning, Guangxi, China; ^3^Division of Genetics and Genomics, Boston Children's Hospital, Harvard Medical School, Boston, Massachusetts, USA

**Keywords:** copy number variant, genetic counseling, pregnancy decision, prenatal cell-free DNA screening

## Abstract

**Background:**

This study explored the associations between positive cell-free DNA (cfDNA) screening results and the decisions made by pregnant women regarding invasive diagnosis and continuation of pregnancy.

**Methods:**

We collected follow-up invasive diagnosis results, pregnancy decisions, and related clinical information for 767 singleton pregnancies with positive cfDNA screening results for common trisomies and genome-wide copy number variants (CNVs) from a cohort of 113,654 singleton pregnancies.

**Results:**

A total of 547 (0.48%) cases of high-chance common trisomies and 220 (0.19%) cases of high-chance CNVs (≥ 3 Mb) were identified through cfDNA screening. The acceptance rate for invasive prenatal diagnosis (IPD) was 89.8% (474/520) in high-chance common trisomies and 75.9% (151/195) in those with high-chance CNVs. The positive predicted value of cfDNA screening was 65.4% for common trisomies (310/474) and 29.1% for CNVs (44/151) in this study. After IPD through SNP array-based chromosomal microarray analysis (CMA), 15.2% (23/151) of high-chance CNVs were classified as pathogenic. Eighty-three percent of pathogenicity (23/24) was observed in concordant high-chance CNVs driven by fetal signals only; 97.1% of parents chose to terminate their pregnancies with confirmed fetal common trisomies, and 95.7% of parents chose to terminate their pregnancies with confirmed pathogenic CNVs.

**Conclusions:**

Currently, the vast majority of cases with positive prenatal cfDNA screening findings underwent IPD. While the technical PPVs were satisfactory, the parental pregnancy choices were largely dependent on the confirmation results. Our findings further demonstrate the clinical utility of prenatal cfDNA screening for CNVs.

## 1. Introduction

Chromosomal abnormalities are present in about one in 150 live births, and it is the reason for nearly half of the early pregnancy losses [1, 2]. An analysis of maternal cell-free fetal DNA (cfDNA) brought the prenatal screening of fetal chromosomal abnormalities into a whole new chapter. cfDNA-based noninvasive prenatal screening (NIPS) was firstly developed for the detection of fetal common autosomal trisomies, that is, Trisomy 21/Trisomy 18/Trisomy 13 (T21/T18/T13), which may cause fetal demise and major congenital disabilities [3]. The clinical application of prenatal cfDNA screening for common autosomal trisomies is well established and recommended as a first-tier screening method by professional committees in recent decades [4, 5]. With technical advancements, cfDNA could also detect genome-wide copy number variants (CNVs), both of benign and pathogenic nature [6]. Fetuses carrying pathogenic CNVs may result in mild to severe congenital malformations, usually with poor prognosis, or even death [7]. Prenatal cfDNA screening is currently the only approach for detecting such CNVs in a noninvasive manner; it is especially critical for cases with pathogenic CNV without overt fetal structural abnormalities indicative of invasive diagnosis [7, 8], yet because of concerns of lack of performance data, generating excess invasive prenatal diagnosis (IPD) and increasing the burden of genetic counseling [9, 10], its routine clinical application remained controversial [11–13].

Here, we present an analysis of 113,654 singleton pregnancies to assess the positive predictive value (PPV) of cfDNA prenatal screening for common trisomies and genome-wide CNVs. Furthermore, we examined parental acceptance towards invasive procedures for CNVs and their choices regarding the continuation or termination of the pregnancy, in comparison to decisions made for common trisomies. Our results offer important insights for genetic counseling in the context of prenatal cfDNA screening for CNVs.

## 2. Materials and Methods

### 2.1. Supervision of the Study

This retrospective clinical research was approved by the Ethics Committee of the Maternal and Child Health Hospital of Guangxi Zhuang Autonomous Region. Enrollments included 113,654 singleton pregnancies from the general population who either opted for or were referred for NIPS at the Maternal and Child Health Hospital of Guangxi Zhuang Autonomous Region from October 2017 to June 2022. All participants had provided informed written consent for their data to be de-identified and used solely for research purposes. Pregnant women with high-chance results underwent posttest counseling and were recommended for invasive prenatal diagnostic testing. The cost of the testing was refundable and covered by insurance, along with NIPS. Seven hundred and sixty-seven samples in our study cohort had a high-chance result for common trisomies or CNVs in NIPS. All of them underwent posttest counseling and were recommended to undergo invasive prenatal diagnostic testing. Follow-up invasive diagnostic results, pregnancy decisions, and related clinical information of pregnancies with NIPS high-chance calls were collected through phone interviews and mailings.

### 2.2. Subjects' Demographics

Between October 2017 and June 2022, 113,654 women with singleton pregnancies underwent NIPS at our clinical center. Out of these, 767 samples tested positive for common trisomies (*n* = 549) and CNV (*n* = 218). The median maternal age for the high-chance common trisomies was 34 years old (ranging from 16 to 48 years old) and 32 years old for the high-chance CNVs (ranging from 15 to 50 years old). In addition, 48.8% of the high-chance common trisomies occurred in women with advanced maternal age (≥ 35 years old, Table 1). Maternal ages of high-chance CNVs are equally distributed among each age group. The median gestational age of patients with high-chance NIPS results was 17 weeks for both types, ranging from 12 to 29 weeks of gestation. NIPS positive pregnancies encountered in this study were mostly achieved through natural conception (Table 1).

### 2.3. NIPS and Bioinformatic Analysis

Five milliliters of peripheral blood was collected from each pregnant woman and stored at room temperature in a cfDNA storage tube (CoWin Bioscience, Jiangsu, China). Wet lab procedures and bioinformatic analysis were performed according to the NIFTY assay (BGI, Wuhan, China) [14]. For each sample, the sequencing raw data generated by BGISEQ500 (BGI, Wuhan, China) was approximately 15 Mb (35 + 10 bp index). A hidden Markov model was introduced to detect and report CNVs larger than 3 Mb [15]. Fetal fraction was calculated using the Y chromosome and a neural network model that incorporates multiple chromosomes [16]. CNVs were identified using the automated bioinformatics analysis system, Halos (BGI, Wuhan, China), which generated a z-score for each identified CNV. Using a validated bioinformatics pipeline to determine the origins of CNVs, CNVs were classified as either fetal or maternal [17]. Fetal CNVs represent CNV calls that are solely driven by signals originating from the fetus, with an absolute z-score of 4 or lower. Maternal CNVs indicate strong signals from the maternal background, exhibiting z-scores with absolute values greater than 4.

### 2.4. Confirmation of NIPS Positive Results

Individuals with a high-chance of common trisomies were confirmed through SNP array analysis (HumanCytoSNAP-12, Illumina) or karyotyping. High-chance CNV cases were confirmed by SNP array or CNV-Seq (Berry, Beijing, China). Confirmatory prenatal diagnosis is primarily conducted through amniocentesis, following the guidelines of the National Health Commission of China. NIPS is only offered to women after 12 weeks of gestation.

### 2.5. Statistical Analysis

The statistical calculations were conducted using IBM SPSS Statistics (Version 22.0, IBM Corp., New York, United States).

## 3. Results

### 3.1. Performance and Diagnostic Rates of NIPS in Detecting Common Trisomies and CNVs

**Figure 1:**
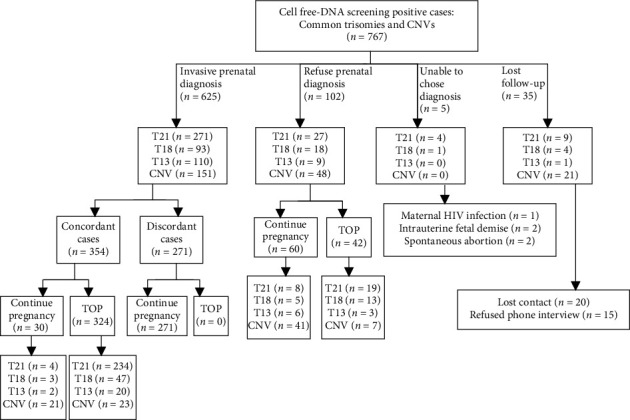
Pregnancy decisions of all prenatal cell-free DNA screening positive cases. TOP, termination of pregnancy. The pregnant woman with HIV infection decided to terminate her pregnancy after receiving a positive cfDNA screening result.

Our study cohort included 113,654 singleton pregnancies that underwent NIPS at our clinical center from October 2017 to June 2022. This yielded a total of 767 calls for high-chance common trisomies (0.48%, *n* = 547) and CNVs (0.19%, *n* = 220). The 547 high-chance common trisomies were composed of T21 (*n* = 311), T18 (*n* = 116), and T13 (*n* = 120, Table 2). Pregnant women with a high chance of T21, T18, and T13 accepted and completed IPD subsequent NIPS at rates of 90.9% (271/298), 83.8% (93/111), and 92.4% (110/119), respectively. In high-chance common trisomies, the cumulative diagnostic rate, that is, the IPD acceptance rate, was 89.8% (474/528). Thirty-five cases of high-chance common trisomies and CNVs lost contact or declined to participate in our follow-up phone interviews. Five cases of high-chance common trisomies were unable to undergo invasive diagnosis due to other reasons, such as being HIV positive, intrauterine fetal demise, and spontaneous abortion (Figure 1). The calculated PPV was 87.8% for T21, 53.8% for T18, and 20.0% for T13. Meanwhile, the study cohort reported 220 cases of high-chance CNV, including 119 cases of fetal CNV and 101 cases of maternal CNVs (Table 2). One hundred and fifty-one of the high-chance CNV pregnancies completed invasive confirmation, resulting in a diagnostic rate of 75.9% (151/199). Diagnostic rates remained the same in subgroups of high-chance fetal CNV (76.6%) and high-chance maternal CNV (75.0%). A total of 44 concordant CNVs were confirmed through invasive diagnosis using SNP array. The calculated PPV was 29.1% in the general high-chance CNVs (44/151), 28.2% in the high-chance fetal CNVs (24/81), and 30.3% in the high-chance maternal CNVs (24/81).

### 3.2. Clinical Significance of NIPS CNVs

**Figure 2:**
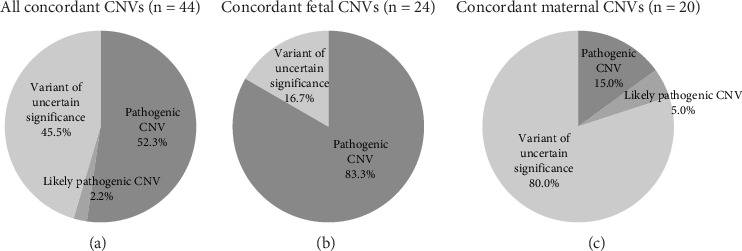
Classifications of concordant copy number variants (CNV) discovered in cell-free DNA screening. Fetal CNVs are high-chance CNVs that are considered to be driven by fetal signals only. Maternal CNVs are high-chance CNVs influenced by maternal signals.

In this study, 148 NIPS CNV positive cases went through SNP array or CNV-Seq confirmation. Among the 44 concordant CNVs, 52.3% were classified as pathogenic (*n* = 23), 2.2% were classified as likely pathogenic (*n* = 1), and 45.5% were classified as variants of uncertain significance (VUS) (*n* = 20, Figure 2a). The pathogenic rate of high-chance CNV was 15.2% (23/151). For fetal signal-driven CNV calls, high-chance fetal CNVs, there were 24 concordant cases: 83.3% of the concordant fetal CNVs were classified as pathogenic (*n* = 20) and 17% were classified as VUS (*n* = 4, Figure 2b). The pathogenic rate in high-chance fetal CNVs was 23.5% (20/85). In 20 concordant cases of maternal CNVs, CNV calls involving maternal background, there were 80.0% VUS (*n* = 16), 15.0% pathogenic variants (*n* = 3), and 5.0% likely pathogenic variants (*n* = 1, Figure 2c). The pathogenic rate for NIPS-maternal CNV was 4.5% (3/66).

### 3.3. NIPS Positive Pregnancies Decision: With and Without IPD

With IPD confirmation, we identified a total of 310 cases of common trisomies (T21 = 238, T18 = 50, T13 = 22), and 301 of them opted to terminate their pregnancies (97.1%) in this study. Two hundred and thirty-four out of the 238 T21 concordant cases (98.3%), 47 out of the 50 T18 concordant cases (94.0%), and 22 out of the 23 T13 concordant cases (95.7%) chose to terminate their pregnancies following IPD confirmation (Table 3). For pregnancies with concordant CNV results, 23 out of the 44 cases chose to terminate their pregnancies (52.3%) and 95.7% of pregnancies with concordant pathogenic CNV terminated pregnancies. The only case of a likely pathogenic CNV, also a maternal signal-driven CNV, decided to continue her pregnancy. Five percent of women with a fetus harboring VUS chose to terminate their pregnancies (Table 3).

For pregnancies that refused further invasive diagnosis, 64.8% of high-chance common trisomies resulted in termination (35/54); 70.4% of high-chance T21, 72.2% of high-chance T18, and 33.3% of high-chance T13 cases that declined invasive confirmation terminated their pregnancies, respectively (Table 4). Out of the 48 cases of high-chance CNV that declined IPD confirmation, only 14.6% (7/48) chose to terminate their pregnancies, while the remaining individuals opted to continue their pregnancies.

## 4. Discussion

It has been clinically accepted and recommended to use cfDNA for NIPS of common autosomal trisomies (T21/T18/T13) [4, 5]. As more studies and evidence emerged, the American College of Medical Genetics and Genomics (ACMG) in 2022 suggested NIPS for detecting 22q11.2 deletion syndrome, and the Dutch Health Council advised routine reporting of high-chance CNVs [13, 18]. Yet whether this approach should be clinically extended to screen for chromosomal abnormalities other than common aneuploidies remains controversial [2, 11, 13]. In the current large cohort-based study, 113,654 singleton pregnancies reported a PPV of 29.1% in CNV calls for variants over 3 Mb. This finding was consistent with other studies that utilized large cohorts and employed a genome-wide sequencing strategy. PPVs of CNVs from previous large group studies ranged from 19% to 50%, depending on the reported variant types and sizes [9, 10, 19]. The positive rate of CNV in NIPS ranged from 0.14% for CNV sizes over 5 Mb to 0.22% for CNV sizes over 2 Mb [9, 20]. Our previous data, which were based on the same platform but had a smaller cohort and only reported CNVs over 5 Mb, yielded a positive rate of 0.16% and PPV of 51.2% [21]. In the present study, we analyzed a cohort that is three times larger than before and identified CNVs over 3 Mb. The positive rate for CNVs was 0.19% and a PPV of 29.1% this time. As the sensitivity level increases, the technique of NIPS usually sacrifices accuracy by producing additional false positive cases. Therefore, when we studied a larger scale cohort with a more precise definition of CNV, it is reasonable to expect an increase in the positive rate and a decrease in PPV. Discordant cases in NIPS may be caused by placental or maternal factors, such as confined placental mosaicism (CPM) or maternal variants and cancers [22]. Vanishing twins and technical factors also contribute, albeit less frequently. A strong correlation was found between CPM and adverse pregnancy outcomes resulting from placental insufficiency [23].

Being the only prenatal robust screening method for fetal CNV, other than PPV, the number of concordant CNV found by NIPS that can be classified as pathogenic in prenatal diagnosis is another practical parameter for genetic counselors. Based on this study, we reported a clinical significance of 15.2% for CNV calls. The clinical significance of high-chance CNVs was found to be consistent with other large population studies of NIPS in detecting fetal microdeletion and microduplication syndrome (MMS), ranging from 13.3% to 15.3% [9, 10]. Interestingly, the studies involving MMS used strategies that narrowed down their CNV definition to 2 Mb at the 22q11.2 region. This was done in order to identify the 22q deletion syndrome, which is a common chromosomal deletion syndrome in fetuses. Since the platform we adopted reports only CNV over 3 Mb, we had one single 22q-related CNV in our concordant cases (Table S1). An upgraded version of the NIPS platform we used in our study, which is compatible with fetal fraction enrichment by nanomagnetic beads [17, 24], will enable us to detect CNV sizes as small as 1.5 Mb in the 22q11.2 area.

Compared to maternal signal-derived high-chance CNV, high-chance fetal CNVs were more likely to pertain to de novo mutations. De novo mutations are exposed to less stringent evolutionary selection; therefore, they are more deleterious and disease-causing than inherited variations [25]. Our results reflected an equivalent scenario. Concordant cases of fetal CNVs had over 83% classified as pathogenic, while a similar proportion of 80% in maternal CNVs concordant cases were classified as VUS (Figure 2). Based on our findings and previous knowledge, understanding the origin of CNV calls is informative in genetic counseling for patients with high-chance CNV results, although an IPD procedure is necessary to confirm the identity of high-chance CNVs.

VUS are genetic changes identified in 1%–3% of chromosomal microarray analyses (CMAs). These variants cannot currently be classified as either benign or pathogenic; however, they may be reclassified in the future, as more information becomes available [26]. VUS introduce probabilistic concepts to parents, and the psychological burden arises when they must make decisions based on these uncertainties. Parental samples may be useful for determining whether CNVs are inherited or de novo. As a method of maternal cfDNA sequencing, NIPS can already indicate whether CNVs are of maternal origin. Inheriting a CNV from an unaffected biological parent may alleviate some concerns; however, it does not eliminate the possibility of disease manifestations in the fetus [27]. Meanwhile, genetic professionals should minimize the reporting of VUS, particularly those with a low Bayesian score, to protect expectant couples from an undue burden [28]. The method of disclosing VUS—whether through direct offer or parental request—should be discussed with couples during pretest counseling [29].

Although uncertain or probabilistic information may impose a psychological burden, it can also hold significant value. Pretest parental choices regarding the disclosure of such information may facilitate the personalized use of advanced genomic tests during pregnancy and afterward, should they choose to continue the pregnancy. Through comprehensive pre- and posttest counseling, expectant parents receive information that addresses their diverse concerns, and the majority express satisfaction with their decisions [30].

Expanding the screening target of NIPS from common trisomies to CNVs will inevitably increase the demand for invasive confirmatory tests. In this study, positive feedback was obtained from a general pregnancy cohort, with 91.5% of participants accepting IPD after receiving high-chance common trisomies results, and 77.1% accepting IPD after receiving NIPS results for high-chance CNVs. These findings indicate a high level of acceptance of IPD in the general population, which is a crucial factor for the clinical implementation of cfDNA screening for CNVs. A similar study conducted in southern China reported a slightly lower acceptance rate of 67% for IPD among NIPS-detected aneuploidies [31]. The high acceptance of IPD in our study may be attributed to two main factors. Firstly, the cost of IPD was covered by insurance for NIPS. Secondly, according to the latest reports and guidelines, the risk of fetal loss associated with amniocentesis is approximately 0.11% [32]. Therefore, healthy pregnant women would be less hesitant to undergo IPD in experienced hospitals. Additionally, pregnant women who choose not to undergo IPD are influenced by key messages provided by genetic counselors when making decisions about their pregnancies. Without IPD, more than 60% of women with NIPS-detected T21 and T18 chose to terminate their pregnancies. In contrast, only 25% of women with NIPS-detected T13 and less than 10% of women with NIPS-detected CNVs made the same choice (Table 4). These termination rates without IPD were consistent with PPVs of the corresponding NIPS targets (Table 2) [9, 10, 33], yet they remained relatively high. This underscores the importance of both pre- and posttest counseling, as well as the potential influence of multimedia in emphasizing the significance of prenatal diagnosis following NIPS. In situations where universal IPD insurance coverage is unavailable, acceptance of IPD may correspondingly decline. Counselors may experience an increased workload, as they need to emphasize the importance of IPD during pre- and post-NIPS counseling sessions. Furthermore, geneticists might consider reporting only selected types of CNVs in NIPS.

## 5. Conclusions

Our study has demonstrated the clinical utility of cfDNA profiling in the detection of both maternal age-dependent common autosomal aneuploidies and maternal age-independent CNVs. The cfDNA screening platform we utilized showed satisfactory performance in identifying pathogenic fetal CNVs. Our findings indicate that IPD is widely accepted among pregnant women in the general population, and effective communication through genetic counseling sessions has been achieved. In other words, the groundwork for the clinical implementation of cfDNA screening for CNVs has been steadily established. Further clinical experience, combined with genetic confirmation of negative cfDNA screening results, along with the development of consensus clinical management and optimal counseling guidelines for diverse populations, may eventually integrate cfDNA screening for CNVs into routine clinical practice. Expanded cfDNA screening combined with ultrasound will set a new benchmark for regular screening of chromosomal abnormalities in pregnancies.

## Figures and Tables

**Table 1:** Demographics of the 767 singleton pregnancies with positive NIPS results.

**Characteristics**	**Common trisomy (** **N** **)**	**Ratio (%)**	**Copy number variant (** **N** **)**	**Ratio (%)**
Maternal age (years)				
< 30	131	23.9	79	36.2
30–34	150	27.3	75	34.4
> 35	268	48.8	64	29.4
Gestational age (weeks)				
1st trimester (6–13)	45	8.2	16	7.3
2nd trimester (14–27)	501	91.3	202	92.7
3rd trimester (> 27)	3	0.5	0	0.0
Conception method				
Natural	527	96.0	209	95.9
Assisted	22	4.0	9	4.1

**Table 2:** Performance of prenatal cell-free DNA screening in fetal common trisomies and copy number variants (CNVs).

**Fetal aneuploidies**	**Positive calls**	**Positive rate (%)**	**Invasive prenatal diagnosis**			**Concordant cases (** **N** **)**	**Discordant cases (** **N** **)**	**PPV (%)**
			**Accept**	**Refuse**	**Diagnostic rate (%)**			
Common trisomy	547	0.48	474	54	89.8	310	164	65.4
Trisomy 21	311	0.27	271	27	90.9	238	33	87.8
Trisomy 18	116	0.10	93	18	83.8	50	43	53.8
Trisomy 13	120	0.11	110	9	92.4	22	88	20.0
All CNVs	220	0.19	151	48	75.9	44	107	29.1
Fetal CNV	119	0.10	85	26	76.6	24	61	28.2
Maternal CNV	101	0.09	66	22	75.0	20	46	30.3

**Table 3:** Pregnancy decisions of cell-free DNA screening positive cases with concordant diagnostic results.

**Prenatal diagnostic results**	**Diagnosed with concordant results**			
	**Total cases (** **N** **)**	**Continue pregnancy (** **N** **)**	**Termination (** **N** **)**	**Termination rate (%)**
Trisomy 21	238	4	234	98.3
Trisomy 18	50	3	47	94.0
Trisomy 13	22	2	20	90.9
Pathogenic CNV	23	1	22	95.7
Likely pathogenic CNV	1	1	0	0.0
Variant of uncertain significance	20	19	1	5.0

**Table 4:** Pregnancy decisions of cell-free DNA screening positive cases declined prenatal diagnosis.

**NIPS results**	**Without prenatal diagnosis**		
	**Continue pregnancy (** **N** **)**	**Termination (** **N** **)**	**Termination rate (%)**
Trisomy 21	8	19	70.4
Trisomy 18	5	13	72.2
Trisomy 13	6	3	33.3
CNV	41	7	14.6

## Data Availability

The data that support the findings of this study are available on request from the corresponding author. The data are not publicly available due to privacy or ethical restrictions.

## Supporting Information

Supporting Information Additional supporting information can be found online in the Supporting Information section. Table S1: cfDNA screening and invasively diagnostic results detail of all concordant cases of high-chance CNVs found by cfDNA screening tests in this study.
