# Supplementary material for: Analysis of Prenatal Diagnosis and Pregnancy Decisions of 767 Singleton Pregnancies With Positive Prenatal Cell-Free DNA Screening Results in Southwest China
Source: J Pregnancy. 2025 Nov 12;2025:8877014. doi: 10.1155/jp/8877014 (PMC12629691; doi:10.1155/jp/8877014)
Supplement: Supporting Information — Additional supporting information can be found online in the Supporting Information section. Table S1: cfDNA screening and invasively diagnostic results detail of all concordant cases of high-chance CNVs found by cfDNA screening tests in this study. [file 8877014.f1.docx]

**Supplementary Table 1 Details of the 44 concordant Copy Number Variants (CNV) found in this study**

|  |  |  |  | SNParray/CNV-Seq | |
| --- | --- | --- | --- | --- | --- |
| Sample Number | Pregnancy weeks | fetal fraction % | NIPS Results | Coordinates of CNVs | Classification |
| 1 | 16 | 9.8 | dup(18p11.32-p11.31,3.84M)-M* | 18p11.32p11.31(680380-3388779)x3 | Variant of Uncertain Significance |
| 2 | 12+5 | 3.8 | del(2q12.2-q13,5.39M)-M | 2q12.2q13(107135170-110982530)x1 | Likely Pathogenic |
| 3 | 14+ | 3.5 | del(18q22.1-q22.2,6.09M)-M | 18q22.1(62437419-66481273)X1 | Variant of Uncertain Significance |
| 4 | 14+1 | 4.7 | dup(16p13.11-p12.3,5.02M)-M | 16p13.11p12.3(15512480-18128488)x3 | Variant of Uncertain Significance |
| 5 | 19 | 8.5 | dup(15q11.2-q13.1,6.13M)-M | 15q11.2q13.1(23667412-28536634)x3 | Pathogenic |
| 6 | 17+4 | 7.6 | del(5p14.2-p13.3,7.46M)-M | 5p14.1p13.3(24713205-31226195)x1 | Variant of Uncertain Significance |
| 7 | 19+4 | 5.6 | del(11p12-p11.2,5.91M)-M | 11p12(38440001-43240000)x1 | Variant of Uncertain Significance |
| 8 | 16+ | 10.7 | dup(4q12-q13.1,5.55M)-M | 4q12q13.1(58193150-62716210)x3 | Variant of Uncertain Significance |
| 9 | 18+2 | 10.8 | dup(2q21.2-q22.2,9.60M)-M | 2q21.2q22.3(133299200-144266489)x3 | Pathogenic |
| 10 | 17+4 | 6.7 | del(4q34.2-q35.1,7.19M)-M | 4q34.3q35.1(178460001-184040000)x1 | Variant of Uncertain Significance |
| 11 | 18+4 | 14.7 | dup(9q21.32-q22.1,5.52M)-M | 9q21.32q21.33(85519130-90013698)x3 | Variant of Uncertain Significance |
| 12 | 16+4 | 11.1 | dup(5q15-q21.2,5.47M)-M | 5q21.1(98425232-101876929)x3 | Variant of Uncertain Significance |
| 13 | 18 | 3.8 | dup(6p12.3-p12.1,7.92M)-M | 6p12.3p12.1(50378802-54910962)x3 | Variant of Uncertain Significance |
| 14 | 16 | 9.0 | dup(21q22.2-q22.3,4.38M)-M | 21q22.3(44585405-45722442)x3 | Variant of Uncertain Significance |
| 15 | 16+6 | 6.6 | del(3q12.3-q13.12,5.05M)-M | 3q12.3q13.11(101012997-104708594)x1 | Variant of Uncertain Significance |
| 16 | 15+2 | 9.0 | dup(4q12-q13.1,6.87M)-M | 4q12q13.1(52728324-65309678)x3~4 | Variant of Uncertain Significance |
| 17 | 17 | 16.8 | del(1q41,5.32M)-M | 1q41(218975899-222195002)x1 | Variant of Uncertain Significance |
| 18 | 22+5 | 9.7 | dup(6p23-p22.3,6.47M)-M | 6p23p22.3(14064273-17778716)x3 | Variant of Uncertain Significance |
| 19 | 16+4 | 14.0 | dup(22q12.1-q12.3,5.10M)-M | 22q12.1q12.3(29083885-35816394)x3 | Variant of Uncertain Significance |
| 20 | 23+ | 9.8 | dup(15q11.2-q13.1,6.13M)-M | 15q11.2q13.1(23222284-29097256)x3 | Pathogenic |
| 21 | 15 | 4.7 | dup(13q21.33-q22.3,9.38M) | 13q21.33q22.1(68928506-73459637)x3 | Variant of Uncertain Significance |
| 22 | 20 | 13.1 | del(7q11.21-q11.23,11.74M) | 7q11.21q11.23(62699114-75553319)x1 | Pathogenic |
| 23 | 16 | 9.7 | dup(2p25.2-p11.2,83.07M) | 2p25.3p11.2(72184-90015508)x3 | Pathogenic |
| 24 | 19+2 | 8.0 | dup(7q31.33-q36.3,33.00M) | 7q32.1q36.3(128357300-159119486)x3 | Pathogenic |
| 25 | 18+2 | 12.5 | del(4p16.3-p15.2,25.04M) | 4p16.3p15.1(891856-3507963)x1 | Pathogenic |
| 26 | 17 | 8.3 | del(5p15.33-p15.1,17.54M) | 5p15.33p15.2(38139-13019834)x1 | Pathogenic |
| 27 | 18 | 6.6 | del(6q13-q16.1,24.68M) | 6q13q16.1(73506104-97476678)x1 | Pathogenic |
| 28 | 17 | 9.5 | del(5p15.33-p15.1,16.53M) | 5p15.33p15.2(568917-14791814)x1 | Pathogenic |
| 29 | 24+ | 22.5 | dup(2q31.1-q33.3,33.01M) | 2q31.2q33.3(175496904-206511442)x3 | Pathogenic |
| 30 | 14+1 | 8.6 | dup(15q11.2-q13.3,9.01M) | 15q11.1q13.3(20161372-32429401)x4 | Pathogenic |
| 31 | 17+1 | 10.3 | dup(9p24.3-p13.1,38.44M) | 9p24.3p12(46587-41966868)x2~3 | Variant of Uncertain Significance |
| 32 | 17+1 | 19.1 | del(5q21.1-q23.1,17.37M) | 5q21.1q23.1(100280001-118780000)x1 | Variant of Uncertain Significance |
| 33 | 17 | 10.5 | del(8p23.3-p12,29.02M) | 8p23.3p23.1(176818-8713038)x1 | Pathogenic |
| 34 | 19+5 | 10.5 | del(7q35-q36.3,12.74M) | 7q34q36.3(141920001-159138663)x1 | Pathogenic |
| 35 | 16+1 | 9.7 | del(9p24.3-p22.1,18.55M) | 9p24.3p22.1(46587-18653380)x1 | Pathogenic |
| 36 | 18+4 | 17.8 | dup(10p15.3-p13,14.90M) | 10p15.3p13(135708-15009109)x3 | Pathogenic |
| 37 | 12+6 | 12.1 | del(8p23.3-p21.2,25.30M) | 8p23.3p23.1(176818-11455106)x1 | Pathogenic |
| 38 | 25+1 | 19.4 | del(22q13.33,3.49M) | 22q13.33(49737239-51169045)x1 | Pathogenic |
| 39 | 17 | 17.3 | dup(12p13.33-p11.21,32.49M) | 12p13.33p11.1(191619-34768168)x3 | Pathogenic |
| 40 | 12+6 | 13.9 | dup(7q21.13-q36.3,68.86M) | 7q21.13q36.3(89843560-159119486)x3 | Pathogenic |
| 41 | 16+4 | 14.3 | del(13q22.2-q34,35.71M) | 13q22.3q34(77256532-114752319)x2 hmz | Variant of Uncertain Significance |
| 42 | 19+5 | 14.8 | del(2q31.1-q32.3,19.26M) | 2q31.1q32.3(181548521-202350955)x1 | Pathogenic |
| 43 | 19+1 | 8.3 | del(4q34.1-q35.2,15.97M) | 4q34.1q35.2(176290024-189667850)x1 | Pathogenic |
| 44 | 12+ | 5.8 | dup(12p13.33-p11.1,33.15M) | 12p13.33p11.1(191619-34768168)x2-4 | Pathogenic |

*The -M after brackets represent maternal CNV.
